# Detailed analysis of clonal evolution and cytogenetic evolution patterns in patients with myelodysplastic syndromes (MDS) and related myeloid disorders

**DOI:** 10.1038/s41408-018-0061-z

**Published:** 2018-03-07

**Authors:** Julie Schanz, Naciye Cevik, Christa Fonatsch, Friederike Braulke, Katayoon Shirneshan, Ulrike Bacher, Detlef Haase

**Affiliations:** 1 Department of Hematology and Medical Oncology, University Medicine Göttingen (UMG), Göttingen, Germany; 2 Department of Dermatology, University Medicine Göttingen (UMG), Göttingen, Germany; 3 Medical University of Vienna, Vienna, Austria; 4 Department of Hematology and Central Laboratory, Inselspital Bern, Bern, Switzerland

## Abstract

Clonal cytogenetic evolution (CE) (i.e., acquisition of new chromosomal aberrations over time) is relevant for the progression of myelodysplastic syndromes (MDS). We performed detailed analysis of CE in 729 patients with MDS and related disorders. Patients with CE showed shorter survival (median OS 18.0 versus 53.9 months; *P* < 0.01), higher leukemic transformation rate (48.0% versus 21.4%; *P* < 0.01) and shorter intervals to leukemic transformation (*P* < 0.01). Two main CE patterns were detected: early versus late CE (median onset 5.3 versus 21.9 months; *P* < 0.01) with worse survival outcomes for early CE. In the case of CE, del (7q)/−7 (*P* = 0.020) and del (17p) (*P* = 0.002) were especially unfavorable. Extending the evolution patterns from Tricot et al. (1985) forming five subgroups, prognosis was best (median OS not reached) in patients with “transient clones/changing clone size”, whereas those with “CE at diagnosis” showed very poor outcomes (*P* < 0.01 for comparison of all). Detailed sequential cytogenetic analysis during follow-up improves prognostication in MDS patients and acknowledges the dynamic biology of the disease. Evidence, time-point, and patterns of cytogenetic clonal evolution should be included into future prognostic scoring systems for MDS.

## Introduction

In around 50% of patients with myelodysplastic syndromes (MDS), clonal cytogenetic abnormalities are detectable by conventional chromosome banding (CCB) from bone marrow metaphases^1–3^ The karyotype is highly significant for prognostication^2–6^ and therapy allocation in patients with MDS and plays a fundamental role for the International Prognostic Scoring System (IPSS)^4^, its revised form, the IPSS-R^6,7^ and the WHO adapted Prognostic Scoring System^8,9^. Molecular mutation screening as well has achieved increasing importance in patients with MDS^10,11^. Therapeutic options in patients with MDS are highly variable including supportive measures, targeted approaches such as lenalidomide in MDS with isolated 5q deletion^12^, and disease-modifying approaches such as azacitidine or decitabine^13,14^. Selected patients with high-risk MDS are candidates for allogeneic hematopoietic stem cell transplantation; so far the only curative approach.

Cytogenetic features are heterogeneous in MDS and are outlined by a preponderance of unbalanced abnormalities. Losses of chromosomal material (e.g., deletion of 5q or 20q, −7, −Y) are more frequent than gains (e.g., +8 or 21)^2,3,15,16^. Complex karyotypes (defined by at least three independent clonal cytogenetic changes)^17^ amount to 15% of all MDS and to 30% of all cytogenetically aberrant cases^2–4^.

Myelodysplastic syndromes are supposed to result from multistep pathogenesis, characterized by increasing malignant potential and clonal evolution^18–20^. This hypothesis suggests a genetic defect on the stem cell level including activation of oncogenes, inactivation of tumor suppressor genes, and alteration of cell cycle regulation^21^. The course of MDS is characterized by chromosome instability and fragility. The sequential acquisition of novel cytogenetic aberrations and the accumulation of these changes result in detectable clonal evolution. Other than that, chromothripsis^22^ occurring in only one catastrophic event leading to chaotic and substantial rearrangements of the chromosome complement can lead to end stage comparable to CE. Clonal evolution may be diagnosed by sequential CCB or by the parallel occurrence of metaphases at different evolution steps in a single cytogenetic analysis. Cytogenetic clonal evolution has been documented in 11.9–39.0% of all MDS cases^3,15,23–26^. Only few studies including 30 to >150 patients^23,25–27^ and one large study (*n* = 988 patients)^28^ investigated progression patterns in patients with MDS. There was no doubt that clonal cytogenetic evolution was associated with a worse prognosis and a more rapid and more frequent transformation to s-AML. Tricot et al^29^. were able to discriminate four clonal cytogenetic evolution patterns: category A referred to MDS patients with a stable clone and low proliferation activity. Patients from category B showed instable clones but low proliferative capacities. Category C referred to patients with stable clones but more pronounced proliferative capacities. Patients from category D showed unstable clones but pronounced proliferative capacities^29^.

We here aimed to perform a most detailed investigation of clonal cytogenetic evolution in a large cohort of 729 patients mostly with MDS and related myeloid disorders such as CMML and s-AML. Within our study we aimed to characterize clonal evolution patterns in detail and to investigate the prognostic impact of the phenomenon of cytogenetic clonal evolution focusing as well on distinct evolution patterns and the complexity of the evolutionary process.

## Material and methods

### Patients

The analysis was based on a total of 729 patients with confirmed MDS (*n* = 478), chronic myelomonocytic leukemia (CMML) (*n* = 63), or s-AML (*n* = 127). The majority (*n* = 701 patients; 96.1%) were adults > 16 years, 28 (3.8%) were pediatric patients. There were 429 males and 300 females (male/female ratio 1.4) with a median age of 65.6 years (range, 1.0–92.2 years). Inclusion in the study was based on the availability of cytogenetic follow-up analyses or detection of cytogenetic clonal evolution (CE) already at diagnosis. The majority of patients (*n* = 663; 90.9%) suffered from de novo disease, 66 patients (9.1%) had therapy-related myeloid disorders (subtypes are shown in Table 1). A total of 472 (64.7%) patients received disease-modifying therapies such as azacitidine or decitabine. Median overall survival of the whole cohort was 37.5 months (corresponding to 3.1 years). Patients had mostly been diagnosed and treated at the University Hospital of Göttingen, Germany; a minority of the samples had been sent from regional hematologic centers. Patients were investigated at the Medical University of Lübeck, Germany (CF and DH) from 1982 to 1993 and at the University of Göttingen, Germany (DH, KS, and JS), from 1993 to 2009.

**Table 1:** Demographic characteristics and cytomorphologic subtypes

Demographic parameters	Number (%)
Total cohort	729 (100.0)
Males: females (ratio)	429: 300 (1.4)
Median age (range), years	65.6 (1.0–92.2)
*Cytomorphologic subtypes*	
Refractory anemia (RA)	212 (29.1)
Refractory anemia with ringed sideroblasts (RARS)	75 (10.3)
Refractory anemia with excess blasts (RAEB)	125 (17.1)
MDS, unclassifiable	61 (8.3)
RAEB in transformation (RAEB-T)	66 (9.1)
s-AML	127 (17.4)
CMML	63 (8.6)

### Methods

CCB was performed on bone marrow cultures according to standard methods by G-bands using a modified GAG-banding technique^3,30^. From 1995 on, metaphases were analyzed with the support of the Ikaros software (Metasystems, Altlußheim, Germany). Karyotypes were documented according to ISCN^17,31^. Selected cases were additionally clarified by 24-color metaphase FISH. In total, 1208 cytogenetic evaluations were performed in the 729 patients. Follow-up analyses were available in 225 patients with 2–10 consecutive analyses per patient. CE was diagnosed when metaphases showing additional clones resulting from CE were detected in parallel to clones without the corresponding aberrations within a single CCB analysis. Alternatively, CE was diagnosed by sequential CCB in case of novel clonal aberrations in addition to the previous aberrations. Complex karyotypes were defined by the simultaneous occurrence of three or more clonal cytogenetic abnormalities.

### Statistical analysis

We used SPSS version 20 (IBM, USA). Overall survival (OS) was calculated according to Kaplan and Meier, comparisons of survival outcomes were performed by log rank test. Patients who were alive at the end of follow-up and patients lost to follow-up were censored. Frequencies were compared by Pearson's chi square, independent variables by Mann–Whitney-*U*. Wilcoxon's test was used for paired samples. Parameters impacting significantly on the probability of survival by univariate analysis were included into multivariate analysis which was performed with the support of proportional hazard regression models. The following parameters were included in the multivariate analysis: sex, age (as continuous parameter), disease-modifying therapy (yes vs. no), hemoglobin ≥ 100 vs. < 100 g/L, neutrophils ≥ 1.8 vs. < 1.8 × 10^9^/L, platelets ≥ 100 vs. < 100 × 10^9^/L, bone marrow blasts (<5% vs. 5–10% vs. 11–20% vs. 21–31% vs. >30%), number of cytogenetic aberrations per case (0 vs. 1 vs. 2 vs. 3 vs. >3), cytogenetic risk score according to the IPSS^4^ (favorable vs. intermediate vs. adverse), cytogenetic risk score according to the IPSS-R^6,7^ (very good vs. good vs. intermediate vs. unfavorable vs. very unfavorable), presence of CE (yes vs. no), CE patterns expanding the criteria of Tricot^29^ as described below (A vs. B vs. C vs. D vs. E). *P*-levels < 0.05 were considered to be significant.

## Results

### Frequency of clonal evolution in the total cohort and within distinct subgroups

CE was detected in 94 patients: in 76 patients at diagnosis, in 18 patients during follow-up. Thus, the frequency of CE was 12.9% (*n* = 94/729) within the total cohort. When only cytogenetically aberrant cases were considered, CE occurred by a frequency of 25.8% (*n* = 92/356). The frequency of CE was higher in females than in males (*n* = 48/300; 16.0% vs. *n* = 46/429; 10.7%; *P* = 0.037). When the different hematologic entities were compared, the frequency of CE was *n* = 66/539 in MDS (12.2%), *n* = 9/63 in CMML (14.3%), and *n* = 19/127 (15.0%) in s-AML. The frequency of CE did not differ significantly between the hematologic entities: MDS versus CMML: *P* = 0.643 (chi square); n.s.; CMML versus s-AML: *P* = 0.902; n.s.; MDS versus s-AML: *P* = 0.409; n.s.

For subsequent analysis, patients were assigned to three groups according to their age at first diagnosis of the MDS (AG1: < 16 years; *n* = 28; 3.9%; AG2: 16–60 years; *n* = 225; 31.4%; AG3: > 60 years: *n* = 464; 64.7%). Twelve patients could not be assigned to a distinct age group (1.7%) as the exact time point of the first diagnosis was not available. CE was more frequent in the higher age groups AG3 and AG2 as compared to AG1 (*n* = 64/464; 13.8% vs. *n* = 27/225; 12.0% vs. 2/28; 7.1%; n.s.). The frequency of CE did not differ significantly between patients with de novo and therapy-related disease (*n* = 84/663; 12.7% vs. *n* = 10/66; 15.2%). When patients were compared for peripheral blood parameters at diagnosis (Supplementary Table 1), there was a non-significant trend to lower neutrophil and thrombocyte counts in patients with CE.

### Prognostic impact of clonal evolution

**Fig. 1:**
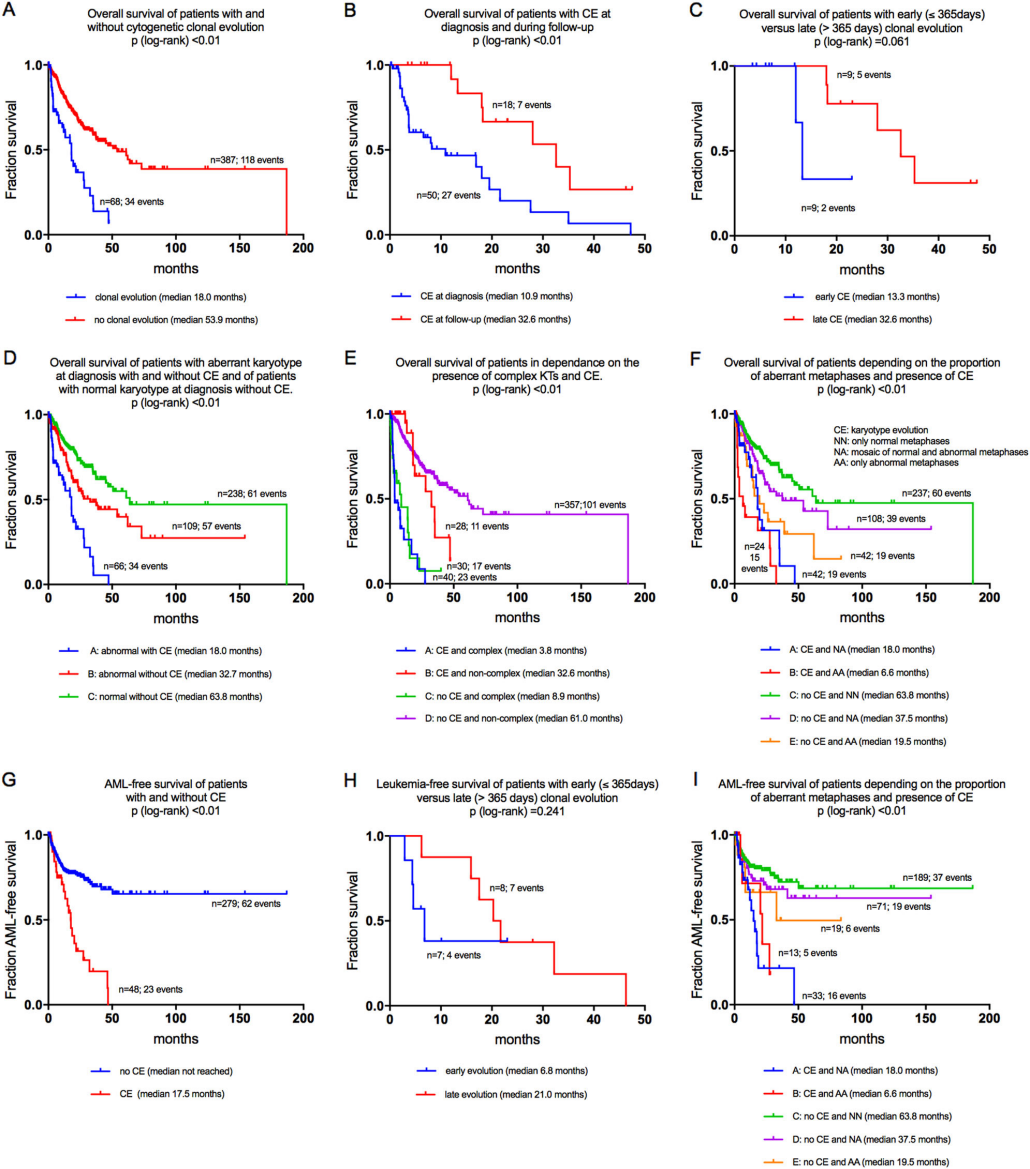
**a**-**i**: Prognostic impact of clonal evolution, time point of evolution and complexity of the karyotype in combination with evolution regarding overall survival and leukemia-free survival

Survival data were available from 68 patients with CE (events 34) and from 387 patients without CE (events 118). OS was shorter in patients with CE than in those without (median 18.0 vs. 53.9 months; *P* < 0.01; Fig 1a). Survival data were available from 50 patients with CE at diagnosis (events 27) and from 18 patients with CE detected during follow-up (events 7). Patients with CE at diagnosis showed a shorter OS than those without (median OS 10.9 vs. 32.6 months; *P* < 0.01; Fig 1b).

Patients with CE at diagnosis showed a higher number of cytogenetic aberrations per case as compared to patients with CE during follow-up of the disease (mean number 4.9; 95%CI: 3.9–5.9; vs. 1.4; 95%CI 0.6–2.1; *P* < 0.01).

### Prognostic impact of the time point of clonal evolution

CE was detected in 18 patients during follow-up by sequential cytogenetic analyses. Survival data were available from all these 18 patients: 11 patients were censored (61.1%); 7 patients had died (38.9%). For separating these 18 patients into two groups according to the time point of occurrence of CE the median interval from diagnosis was chosen as threshold. The median interval was 363 days. In order of practicability, we thus chose a threshold of 365 days for the respective separation: Patients with CE within the first 365 days from diagnosis were classified as “early CE” (*n* = 9), those with CE occurring later than 365 days from diagnosis as “late CE” (*n* = 9). Within the subgroup of patients with “early CE” (less than 1 year from diagnosis), the median time point of detection of CE was 5.3 months (range, 2.8–10.1 months). The subgroup with “late CE” (at more than 1 year from diagnosis) was developing CE after a median of 21.9 months from diagnosis within a range from 13.8 to 46.3 months. Thus, different kinetics of CE became obvious. Patients with early CE showed shorter OS than those with late CE (median 13.2 vs. 32.6 months; *P* = n.s.; Fig 1c). Although significance was not reached (probably due to the limited case number) this difference in the survival outcomes of both subgroups as discriminated by the time point of occurrence of CE suggests a higher level of cytogenetic instability in the patients with early CE.

### Cytogenetic aberrations

Patients with CE more frequently had aberrant karyotypes at diagnosis than those without CE (*n* = 92/94; 97.9% vs. *n* = 262/633; 41.4%; *P* < 0.01).

We performed comparison of survival outcomes depending on the presence of aberrant karyotypes at diagnosis and depending on the occurrence of CE. For this comparison, only three subgroups could be compared as only two patients initially showed a normal karyotype and developed CE during follow-up. Patients with a normal karyotype at diagnosis without CE showed the best median OS of 63.8 months. Patients with aberrant karyotypes at diagnosis without CE showed a median OS of 32.7 months, those with aberrant karyotypes at diagnosis and presence of CE at the time of diagnosis had a median OS of 18.0 months only and thus were representing the most unfavorable subgroup (*P* < 0.01 for comparison of all subgroups and for all single comparisons; Fig 1d).

### Cytogenetic complexity

Cytogenetic complexity was categorized according to the number of clonal cytogenetic aberrations at diagnosis following the suggestion of Heim and Mitelman^32^: group 1—no cytogenetic aberration; group 2—one clonal aberration; group 3—two clonal aberrations, group 4—three clonal aberrations, group 5—more than three clonal aberrations. Normal karyotypes at the time point of cytogenetic analysis were rare in patients with CE as compared to the frequency in patients without CE (*n* = 2/94; 2.1% vs. 371/633; 58.6%; *P* < 0.01). Presence of one clonal aberration was more frequent in the patients without CE, whereas presence of 2, 3, and >4 clonal aberrations at diagnosis was more frequent in patients with CE (*P* for comparison of all, *P* < 0.01; Table 2). Complex karyotypes (≥3 clonal abnormalities) were found more frequently at diagnosis in patients with CE than in patients without: 50 of 92 patients (54.3%) with CE already at diagnosis or during follow-up vs. 57/264 (21.6%) patients without CE (*P* < 0.01). Thus, the phenomenon of CE was associated with higher cytogenetic complexity.

**Table 2:** Cytogenetic complexity at diagnosis in patients with and without CE

No. of aberrations per case	No. (%)		*P*-value (Chi²)
	Pts without CE	Pts with CE	
0	371 (58.6%)	2 (2.1%)	<0.01
1	177 (28.0%)	13 (13.8%)	
2	25 (3.9%)	29 (30.9%)	
3	16 (2.5%)	9 (9.6%)	
>3	44 (7.0%)	41 (43.6%)	

Focusing on patients with CE, median OS of patients with complex karyotypes at diagnosis was 3.8 months as compared to 32.6 months for patients with non-complex karyotypes at diagnosis (*P* < 0.01). In the subgroup of patients with complex karyotypes, patients with CE showed a median OS of 3.8 months as compared to 8.9 months in those without (*P* < 0.01; Fig 1e).

### Size of aberrant clones

The finding of aberrant metaphases only (i.e., all metaphases analyzed aberrant) at diagnosis was more frequent in cases with CE than in those without (38/94; 40.4% vs. 77/623; 12.4); mosaic karyotypes (meaning the parallel occurrence of aberrant and normal metaphases) were found in 54/94 (57.4%) of cases with CE as compared to 176/623 (28.3%) in patients without (*P* < 0.01). Patients with CE showed a htranigher mean proportion of 84.8% of aberrant metaphases at diagnosis as compared to 72.9% in patients without CE (*P* < 0.01). In the subgroup of patients with aberrant metaphases only, patients with CE showed shorter survival than those without (median OS 6.6 vs. 19.5 months; *P* = 0.022; Fig 1f). Considering only mosaic karyotypes, survival of patients with CE was significantly shorter as compared to patients without (median OS 18.0 vs 37.5 months; *P* = 0.009).

### Leukemic transformation

Transformation to s-AML was more frequent in patients with CE as compared to patients without CE (24/50; 48.0% vs. 67/313; 21.4%; *P* < 0.01). Patients without CE showed a plateau of the LFS, whereas all patients with CE developed s-AML. The median LFS was 17.5 months in patients with CE, whereas it was not reached in the subgroup without CE (Fig 1g). The LFS in the subgroup with early CE (<365 days from diagnosis) was 6.8 months only in contrast to 21.0 months in patients with late CE (occurring more than 365 days from diagnosis; *P* = 0.241; Fig 1h). Considering only patients with aberrant metaphases only, those with CE showed a median LFS of 6.6 months as compared to 19.5 months in those without (*P* = <0.01). When only patients with mosaic karyotypes were considered, median LFS was 18.0 months in those with CE whereas median LFS was 37.5 months in those without (*P* < 0.01; Fig 1I).

### Characterization of involved chromosomes

Most frequently, CE was resulting from aberrations of chromosomes 5 (*n* = 51), 7 (*n* = 35), and 8 (*n* = 27). This is shown in more detail in Supplementary Figure 1. Subsequently, distinct chromosomes/chromosomal regions were considered, in particular 5q deletion, −7/del(7q), +8, 17p abnormality, 20q abnormality, and −Y.

**Fig. 2:**
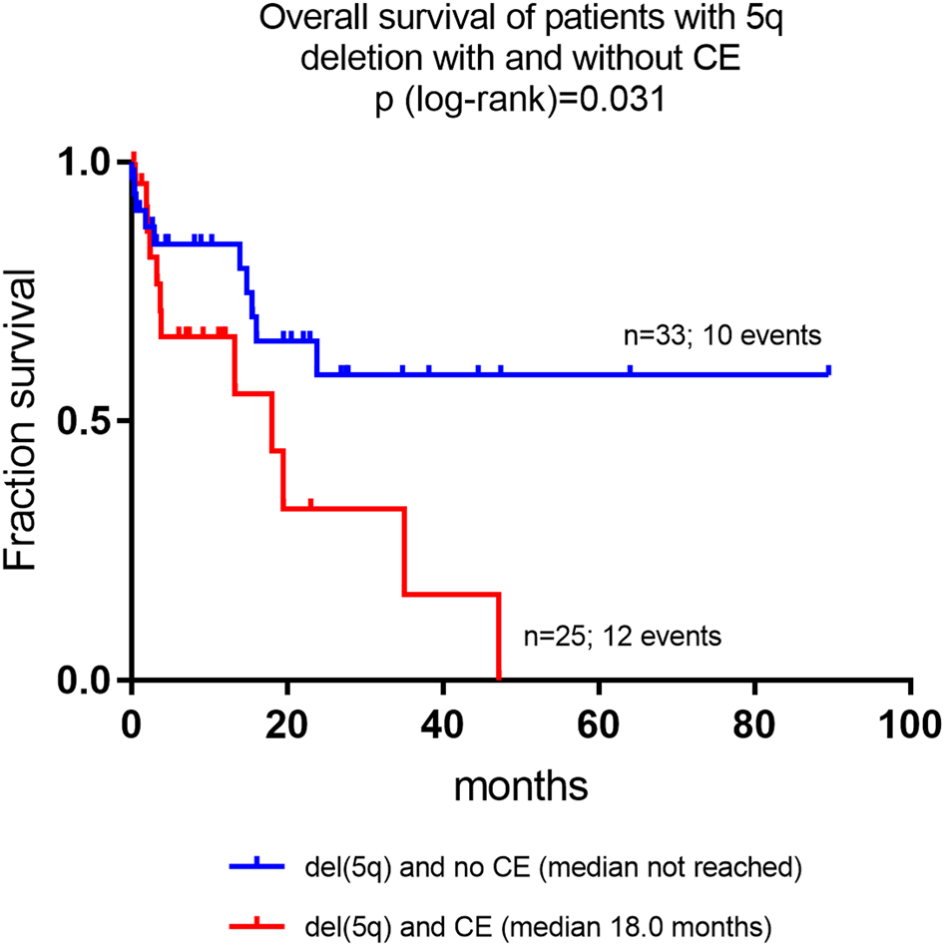
Overall survival of patients with 5q-deletion with and without clonal evolution

Patients with CE showed a higher frequency of del(5q) at diagnosis as compared to those without (43/92; 46.7% vs. 66/198; 25.0%; *P* < 0.01). Within the subgroup of patients with del(5q) at diagnosis, survival data were available from 25 patients with CE (events 12) and from 33 patients without CE (events 10). Patients with del(5q) and CE showed a median OS of 18.0 months whereas median OS was not reached for those with del(5q) without CE (*P* = 0.031, Fig 2). Within the subgroup of patients with CE, the median OS did not differ significantly between those with del(5q) and those without del(5q) (13.3 vs. 18.2 months; n.s.).

Chromosome 7 aberrations—i.e., −7/del(7q)—were detected in 88 out of all 356 patients with aberrant karyotypes at diagnosis (24.7%). At diagnosis, they did not differ significantly between patients with CE and those without (*n* = 27/92 patients; 29.3% vs. 61/264 patients; 23.1%). Survival data were available from 72 patients with CE (of those, 20 patients with chromosome 7 aberrations). Within patients with CE, the median OS of patients with −7/del(7q) was worse as compared to patients without any chromosome 7 aberration (8.2 vs. 18.2 months; *P* = 0.020).

Trisomy 8 was detected in 65/356 cases (18.2%) with aberrant karyotypes with a similar frequency in both subgroups (i.e., patients with CE and patients without; *n* = 19; 20.7% vs. *n* = 46; 17.4%; n.s.). Survival data were available in 13 cases with CE and +8 (censored in 9 cases) and in 59 patients without +8 (censored in 31 cases). Considering only patients with CE, median OS did not differ significantly between those with +8 and those without +8 (median OS not reached vs. 17.9 months; n.s.).

A total of 8 patients (2.2%) were affected by 17p deletions. 17p deletions were more frequent in patients with evidence of CE than in cases without (*n* = 5; 5.4% vs. *n* = 3; 1.1%; *P* = 0.030). When only patients with CE were considered, patients with del(17p) showed a shorter median OS of 1.8 months as compared to patients without del(17p) (1.8 vs. 17.9 months; *P* = 0.002).

20q deletion was detected in patients with and in patients without CE by a similar frequency (*n* = 9; 9.8% vs. *n* = 15; 5.7%; n.s.). Within the subgroup of patients with CE, the median OS of patients with del(20q) and of those without del(20q) did not differ significantly (17.9 months vs. 16.9 months; n.s.).

Loss of the Y chromosome was identified in male patients with CE and those without by a similar frequency (*n* = 4; 4.3% vs. *n* = 14; 5.3%; n.s.). Survival data were available from 72 patients, only 3 of which showed −Y, which was not sufficient for statistical calculation.

## Cytogenetic evolutionary patterns

Extending the criteria of Tricot et al^29^. to a cytogenetic basis five follow-up patterns were defined for this study: A. “Genetic progression”: cases with an initially normal karyotype gaining additional aberrations during follow-up, cases showing an initially abnormal karyotype gaining further aberrations during follow-up, cases with an increase of clone size by >20% and cases initially showing a non-complex karyotype but later transforming into a complex karyotype. Thus, this category refers to patients with the phenomenon of CE during follow-up but also to patients who show genetic progression but no sign of CE as defined above. B. “Genetic stability”: patients with an unchanged normal or an unchanged aberrant karyotype (patients without any sign of CE). C. “Genetic regression”: patients with an aberrant karyotype at the initial analysis achieving a reduction of the aberrant clone of more than 20% or transforming to a normal karyotype during follow-up (possibly related to disease-modifying therapy). D. “CE at diagnosis”: patients with different but depending clones showing additional clonal chromosomal aberrations in parallel to the stem clone. E. “Transient clones/changing clone size”: patients with transient clones that appear only temporary or patients that show an undulant clone during the course of the disease.

We were able to investigate 280 patients for these cytogenetic evolution patterns: A total of 76 patients showed CE already at diagnosis of the MDS; for 21 of those cytogenetic follow-up investigations were available, whereas 55 patients were investigated only once at diagnosis. In addition, cytogenetic follow-up investigations were available in another 204 patients from the original cohort. The largest subgroup was identified for pattern B/“genetic stability” in 126 patients (45.0%). This was followed by pattern A/“genetic progression” in 73 patients (26.1%); pattern D/“CE already at diagnosis”: *n* = 55 (19.6%); pattern C/“genetic regression”: *n* = 18 (6.4%). The smallest subgroup was represented by pattern E/“transient clones/changing clone size”: *n* = 8; 2.9%).

When patients were investigated for the frequencies of complex karyotypes within these cytogenetic evolution patterns, these were most frequent in the patients with CE at diagnosis (pattern D) with 36/54 (66.7%) of patients. Frequencies of complex karyotypes were detected in the other subgroups as follows: genetic progression (pattern A): *n* = 6/54 (11.1%); genetic stability (pattern B): *n* = 9/54 (16.7%); “genetic regression” (pattern C): *n* = 3/54 (5.5%); transient clones/changing clone size (pattern E): *n* = 0/54 (0.0%).

### Cytogenetic aberrations within clonal evolution

We specifically focused on the 18 patients in whom CE was observed during follow-up. The most frequent primary clonal cytogenetic aberrations at first diagnosis were: del(5q): *n* = 4; −7/del(7q): *n* = 3; del(20q): *n* = 2. Clonal evolution during follow-up was most frequently characterized by the occurrence of chromosome 7 aberrations—i.e., −7/del(7q)]—or +8 (*n* = 3 cases each). An additional –Y was detectable in 2 of these 18 cases. One case with a normal karyotype at diagnosis proceeded to a complex karyotype. Another case with a normal karyotype at diagnosis developed del(5q) and −21. Supplementary Table 2 shows the karyotypes of patients with CE at diagnosis and during follow-up.

### Occurrence of distinct aberrations within different clonal evolution patterns

Patients with pattern D/CE at diagnosis showed a high frequency of del(5q) (*n* = 32; 58.2%), −7/del(7q) (*n* = 16; 29.1%), and +8 (*n* = 11; 20.0%). 17p aberrations were found in four cases (7.3%). Patients with pattern A/“genetic progression” showed −7/del(7q) and del(5q) in 12 cases (16.4%) each. Trisomy 8 was detected in 8 cases (11.0%). Del(20q) (*n* = 6; 8.2%) was less frequent. Patients with pattern B/“genetic stability” showed most frequently +8 (*n* = 10; 7.9%). Patients with pattern C/“genetic regression” frequently showed −7/del(7q) (*n* = 5; 27.8%). In patients with pattern E/“transient clones/changing clone size”, del(5q) was found in three cases (37.5%). −7/del(7q), del(20q) and 17p aberrations were not detectable. Thus, −7/del(7q) was occurring in all evolution patterns but “transient clones/changing clone size” (pattern E). Supplementary Figure 2 illustrates associations between distinct chromosomal aberrations and the different clonal evolution patterns.

### Prognostic profiles according to clonal evolution patterns

**Fig. 3:**
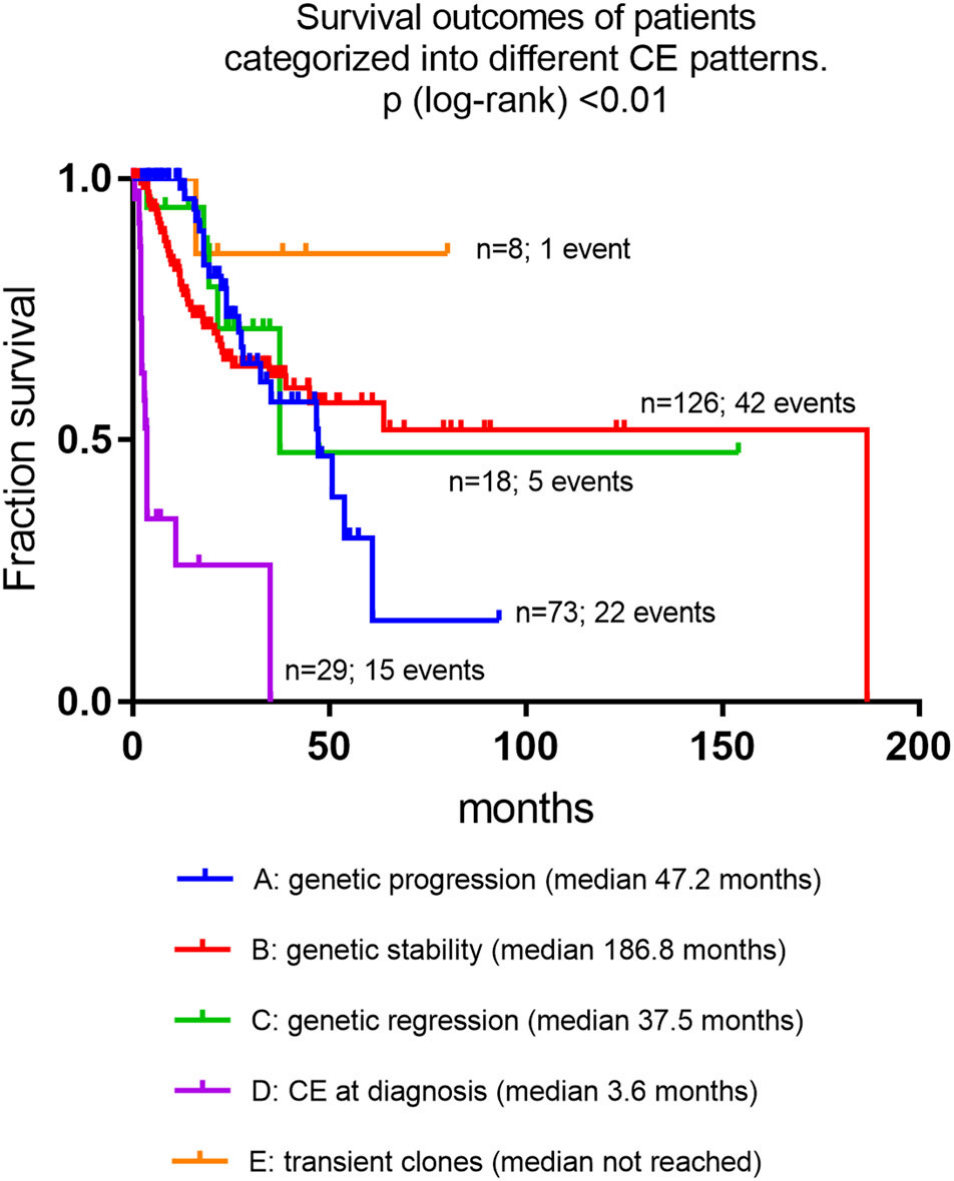
Overall survival of patients categorized into different clonal evolution patterns

Prognosis was best with median OS not being reached in patients with pattern E/“transient clones/changing” clone size. Patients with pattern B/“genetic stability” showed a very favorable median OS of 186.8 months, patients with pattern A/“genetic progression”: 47.2 months, pattern C/“genetic regression”: 37.5 months. Those with pattern D/“CE at diagnosis” showed median OS of 3.6 months only (*P* < 0.01 for comparison of all subgroups; Fig 3).

### Univariate and multivariate analysis

Supplementary Table 3 shows the results of uni- and multivariate analysis for prognostically relevant parameters. By univariate analysis for parameters impacting on the OS, patients with CE showed a 2.7-fold increased HR (*P* < 0.01). When patients were grouped according to the peripheral blood thresholds given by the IPSS^4^, hemoglobin < 100 g/L (*P* < 0.01), neutrophils < 1.8 × 10^9^/L (*P* = 0.013), and thrombocytes < 100 × 10^9^/L (*P* < 0.01) were associated with a higher HR. Increased bone marrow blasts were also associated with a higher HR (*P* < 0.01). Presence of CE was linked with a HR of 2.7 (*P* < 0.01). Patients with pattern D/“CE already at diagnosis” had a HR of 11.3 (*P* < 0.01) as compared to the reference group pattern A/“genetic progression”. Presence of 3 and of >3 clonal aberrations was associated with increased HRs of 6.7 and 9.4 (*P* < 0.01 for both). Also the cytogenetic risk groups according to the IPSS^4^ and the IPSS-R^6,7^ significantly impacted on OS. Age and sex showed non-significant trends only. Application of disease-modifying treatment showed no significant impact on OS.

All above parameters but the cytogenetic risk groups according to IPSS and IPSS-R were included into multivariate analysis for parameters impacting on the OS (Supplementary Table 3). Higher bone marrow blasts maintained their significant adverse impact on OS. Presence of three clonal cytogenetic aberrations was associated with a HR of 5.7, presence of >3 aberrations with a HR of 8.3 (*P* < 0.01 for both). Presence of CE was associated with an increased HR of 3.6 (*P* = 0.013). Patients with “genetic stability” and “CE already at diagnosis” had a higher risk of mortality (HR for pattern B: 2.0; for pattern D: 1.9; *P* = n.s.). Patients with “genetic regression” and those with transient clones/changing clone size showed a reduced risk of mortality (HR for pattern C: 0.4; for pattern E: 0.6; *P* = n.s).

## Discussion

In this study, we evaluated the prognostic impact of CE and of distinct clonal evolution patterns at diagnosis and during follow-up in 729 patients with MDS and related disorders (CMML and s-AML). So far, only few studies were focusing on this issue in larger patient cohorts. Tricot et al. investigated progression patterns including cytogenetic changes and clinical profiles in 46 patients^29^. Some more authors were investigating cohorts with <50^24,33,34^ or <100 patients^23,26^. The largest cohorts with 100–200 patients were published by Geddes et al^27^. and White et al^15^. Haferlach et al. presented data of 988 patients at an international conference but did not differentiate distinct CE patterns^28^. At present, molecular evolution patterns are being evaluated in patients with MDS by means of high-throughput sequencing ^35^.

The overall frequency of cytogenetic CE was 12.9% in our cohort, and we found an association of CE with male gender. When only cases with aberrant karyotypes were considered, the frequency of CE was 25.9% which was in the range of the previous literature (documenting CE in a range between 10 and 40%) 15, 24 This range may be due to heterogeneous patient cohorts and varying follow-up periods in the different studies.

We found CE to be clearly associated with an adverse prognosis with a median OS of 18.0 months only in patients with CE in contrast to 53.9 months in those without (*P* < 0.01). This was in confirm of previous data describing more adverse survival outcomes in patients with CE as compared to those without^23,26,27^. Beyond that, we were able to show for the first time that the time point of CE is also relevant. Patients with CE already at diagnosis showed an inferior median OS of 10.9 months as compared to 32.6 months in patients developing CE during follow-up (*P* < 0.01). As CE at diagnosis was detected due to the parallel occurrence of different clones at initial investigation, these patients may have already developed advanced disease with several steps of cytogenetic evolution before the clinical manifestation which triggered the investigation for and the diagnosis of the respective myeloid malignancy. Interestingly, we were able to discriminate different dynamics of CE during follow-up. Patients developing CE during the first year from diagnosis showed more adverse survival outcomes than those developing CE at >12 months. This suggests that occurrence of CE at an earlier time point may be associated with higher levels of genetic instability. A significant association between CE and rapid leukemic transformation had been demonstrated also in previous studies ^23,26,27,29,36^.

The occurrence of distinct chromosome abnormalities within CE, i.e., of chromosomes 7 or 17p, was associated with a significantly shorter OS. Thus, the types of chromosomal abnormalities occurring during the process of CE are also relevant.

The frequency of s-AML transformation was higher with 48.0% in cases with CE as compared to 21.4% in those without in our study, and the LFS was significantly shorter (*P* < 0.01). Bernasconi et al. found a 36-fold increased risk of leukemic transformation in patients with CE^25^. Progression of MDS is considered to be mediated by stepwise acquisition of novel clonal aberrations^18–20^. In our study, a higher number of cytogenetic aberrations at diagnosis showed significant association to the presence of CE. In patients with CE, those with complex karyotypes showed significantly worse survival outcomes. Furthermore, complex karyotypes showed a double as high frequency in patients with CE as compared to those without. Similarly, Haferlach et al. demonstrated an association of CE with an increase of cytogenetic complexity^28^ which also correlates with higher risk IPSS and IPSS-R scores. Increase of the clonal size had been shown to be prognostically relevant in MDS patients^15,27,37^. In our study, patients with CE with a 100% clonal size (only aberrant metaphases traceable) had a significantly worse OS as compared to those with CE affecting only part of the metaphases.

The most frequent primary aberrations in cases developing CE were del(5q), −7/del(7q), del(20q), and –Y in our cohort which was corresponding to the well-known frequent occurrence of the respective abnormalities in MDS^4,6,7^. Similarly, Jabbour et al. had described chromosome 7 aberrations in 20% and trisomy 8 in 15% of patients with CE in a lower risk MDS cohort^38^. Thus, we were able to confirm previous studies regarding primary and secondary aberrations in patients with MDS^39–41^. The overall spectrum of chromosomal abnormalities in the cases with CE in our study was large including, e.g., rare numerical aberrations such as −22. Finally, −7/del(7q) was prognostically unfavorable when occurring within CE (*P* = 0.020) in analogy to the adverse prognostic impact of chromosome 7 aberrations ^6,7^.

Extending and modifying the criteria of Tricot et al^29^. to a merely cytogenetic categorization, the best survival was observed in patients with “transient clones/changing clone size”, whereas those with “CE already at diagnosis” had very poor outcomes. One may only speculate that transient changes have less significance as they may correlate to only transient periods of cytogenetic instability. Also, disappearance of pre-existing clones could be indicative for presence of a more competent immuno-surveillance. Considering the limited size of these cytogenetic subgroups further investigation of these associations by independent study groups is desirable.

In conclusion, we were able to confirm cytogenetic clonal evolution to represent an independent adverse prognostic factor for patients with MDS and related myeloid disorders. Furthermore, our study emphasizes the value of sequential cytogenetic monitoring during follow-up of MDS for detecting CE. The detection of CE already at diagnosis is prognostically more adverse as compared to its occurrence during the later follow-up. Current risk stratification systems do not focus on disease dynamics in MDS^4,7^. According to our results and previous studies, MDS research should focus on integrating clonal cytogenetic evolution and the different clonal evolution patterns into already established risk stratification systems in the near future.

## Electronic supplementary material

Supplementary Table 1

Supplementary Table 2

Supplementary Table 3

Supplementary Figure 1

Supplementary Figure 2
