# Supplementary material for: Detailed analysis of clonal evolution and cytogenetic evolution patterns in patients with myelodysplastic syndromes (MDS) and related myeloid disorders
Source: Blood Cancer J. 2018 Mar 7;8(3):28. doi: 10.1038/s41408-018-0061-z (PMC5841340; doi:10.1038/s41408-018-0061-z)
Supplement: Supplementary file 1 — Supplementary Table 1 [file 41408_2018_61_MOESM1_ESM.docx]

**Supplemental Table T1:** Comparison of peripheral blood values and marrow blasts in patients with and without cytogenetic clonal evolution (CE)

(BM: bone marrow; ANC: absolute neutrophil counts; MWU: Mann-Whitney U test)

| **Parameter** | **Whole cohort, no (%)** | **Patients with CE, no (%)** | **Patients without CE, no (%)** | ***P-value*** |
| --- | --- | --- | --- | --- |
| **Valid** | 538 (100.0) | 72 (100.0) | 466 (100.0) |  |
| **BM blasts (%)** |  |  |  | Chi^2^ |
| <5 | 285 (53.0) | 32 (44.4) | 253 (54.3) | 0.589 |
| 5–10 | 85 (15.8) | 12 (16.7) | 73 (15.7) |  |
| 11–20 | 57 (10.6) | 10 (13.9) | 47 (10.1) |  |
| 21–30 | 32 (5.9) | 5 (6.9) | 27 (5.8) |  |
| >30 | 79 (14.7) | 13 (8.1) | 66 (14.2) |  |
| **Hemoglobin, g/L** |  |  |  | MWU |
| Median (range) | 96 (47-164) | 94 (52-129) | 97 (47-164) | 0.29 |
| **Leukocytes, ×10^9^/L** |  |  |  |  |
| Median (range) | 3.7 (0.5-170) | 3.7 (1.2-170) | 3.6 (0.5-138.1) | 0.7 |
| **ANC, ×10^9^/L** |  |  |  |  |
| Median (range) | 1.5 (0-20.6) | 0,6 (0.6-4) | 1.7 (0.2-20.6) | 0.221 |
| **Thrombocytes, ×10^9^/L** |  |  |  |  |
| Median (range) | 89.5 (0-966) | 69 (11-521) | 96 (0.0-966) | 0.133 |
