# Supplementary material for: Detailed analysis of clonal evolution and cytogenetic evolution patterns in patients with myelodysplastic syndromes (MDS) and related myeloid disorders
Source: Blood Cancer J. 2018 Mar 7;8(3):28. doi: 10.1038/s41408-018-0061-z (PMC5841340; doi:10.1038/s41408-018-0061-z)
Supplement: Supplementary file 2 — Supplementary Table 2 [file 41408_2018_61_MOESM2_ESM.docx]

| **Pat No** | **Karyotype at diagnosis** | **Karyotype at the time of CE** |
| --- | --- | --- |
| 82 | 47,XX,t(1;3)(p36.1;q21),+21[25] | 47,XX,t(1;3)(p36.1;q21),+21[18]/47,idem,add(17)(p12)[4] |
| 218 | 45,XY,-7[7] | 45,XY,-7[?]/45,idem,t(2;11)(q31;p15)[?]/46,XY[?] |
| 227 | 47,XY,+i(1)(q10)[26]/  46,XY[4] | 47,XY,+i(1)(q10)[?]/47,idem,del(20)(q11.2)[?] |
| 247 | 46,XY,+1,der(1;7)(q10;p10)[39]/ 46,XY[5] | 46,XY,+1,der(1;7)(q10;p10)[?]/47,idem,+8[?] |
| 264 | 45,X,-Y[18] | 45,X,-Y[13]/45,idem,del(7)(q22)[5] |
| 447 | 46,XX[20] | 47,XX,der(6)t(1;6)(q21;p11),+8[4]/  47,XX,der(6)t(1;6)(q21;p11),+19[2]/  47,XX,+8,der(17)t(1;17)(q21;p13)[11]/  47,XX,der(17)t(1;17)(q21;p13),+19[19]/  46,XX[3] |
| 552 | 46,XX,del(5)(q13q31)[20]/  46,XX[5] | 46,XX,del(5)(q13q31)[22]/47,idem,+4[3] |
| 584 | 46,XY,del(7)(q11q35)[8]/  46,XY[6] | 46,XY,del(7)(q11q35)[18]/45,idem,-22[6] |
| 602 | 47,XX,+8[3]/46,XX[22] | 47,XX,+8[7]/48,idem,+21[10]/46,XX[4] |
| 686 | 46,XX[14] | 46,XX,del(5)(q13q31)[7]/45,idem,-21[2]/46,XX[7] |
| 691 | 46,XY,del(5)(q13q33)[4]/  46,XY[1] | 46,XY,del(5)(q13q33)[1)/  46,idem,t(2;11)(p21;q23)[1]/  46,XY[23] |
| 693 | 46,XY,del(20)(q11.2)[1] | 46,XY,del(20)(q11.2)[3]/47,idem,+8[3]/  46,idem,-Y,+8[13]/46,XY[4] |
| 749 | 46,XY,del(20)(q11.2q13.3)[19]/  46,XY[7] | 46,XY,del(20)(q11.2q13.3)[10]/45,idem,-7[15] |
| 755 | 46,XY,del(5)(q13q33)[15]/  46,XY[10] | 46,XY,del(5)(q13q33)[2]/  46,idem,del(16)(q12.1)[13]/46,XY[11] |
| 772 | 45,XY,del(3)(q21~23q26.2),-7[25] | 45,XY,-7[8]/45,idem,t(1;3)(q24;q26.1)[12]/  46,XY[2] |
| 781 | 46,XY,del(5)(q13q33)[4]/  46,XY[21] | 46,XY,del(5)(q13q33)[11]/  45,idem,del(8)(q22),der(12)t(12;20)(p12;?),der(13)  t(13;18)(p11;q?),del(18)(q12),-20[3]/46,XY[11] |
| 800 | 46,XY,der(3)(q?)[4]/46,XY[2] | 43,X,-Y,der(5)t(5;16)(q?22~31;?),-7,-16,del(17)(p13),der(19)t(16;19)(?;?q11)[cp5]/  44,X,-Y,der(1)t(1;3;6)(p?;?;p25),der(15)t(15;18)(?;q?),-18,der(19)t(16;19)(?;?q11)[cp6]/46,XY[3] |
| 806 | 41~45,XX,dic(1;14)(p36.3;p13),-5,add(6)(p25),add(9)(q34),  t(11;15)(q12;p13),  -15,add(16)(p13),  +1~2mar[cp10]/46,XX[21] | 45,XX,dic(1;14)(p36.3;p13),-10,-16,+2mar[13]/  44,XX,dic(1;14)(p36.3;p13),add(7)(p22),-10,  -16,+mar[2]/46,XX[1] |

Supplemental Table T2: Detailed karyotypes at diagnosis and at the time of cytogenetic clonal evolution (CE) in 18 patients from the study
