# Supplementary material for: Detailed analysis of clonal evolution and cytogenetic evolution patterns in patients with myelodysplastic syndromes (MDS) and related myeloid disorders
Source: Blood Cancer J. 2018 Mar 7;8(3):28. doi: 10.1038/s41408-018-0061-z (PMC5841340; doi:10.1038/s41408-018-0061-z)
Supplement: Supplementary file 3 — Supplementary Table 3 [file 41408_2018_61_MOESM3_ESM.docx]

| **Supplemental table T3: Results from uni- and multivariate analyses** | | | | | | | | | | | | | |
| --- | --- | --- | --- | --- | --- | --- | --- | --- | --- | --- | --- | --- | --- |
|  | | **Univariate, OS** | | | | **Multivariate, OS** | | | | **Multivariate, LFS** | | | |
| **Parameter** | **category** | **n** | **HR** | **95% CI** | **p** | **n** | **HR** | **95% CI** | **p** | **n** | **HR** | **95% CI** | **p** |
| **Sex** | male | 300 | 1,3 | 0,9-1,8 | 0,061 | 127 | 1,4 | 0,7-2,6 | 0,328 | 112 | 1,6 | 0,8-3,2 | 0,148 |
|  | female | 193 |  |  |  | 75 |  |  |  | 62 |  |  |  |
| **Age** |  |  | 0,9 | 0,9-1,0 | 0,065 |  | 1,0 | 1,0-1,0 | 0,616 |  |  |  |  |
| **Therapy** | no | 105 | 0,7 | 0,5-1,1 | 0,099 | 8 | 0,6 | 0,1-4,8 | 0,646 | 6 |  |  | 0,978 |
|  | yes | 388 |  |  |  | 194 |  |  |  | 168 |  |  |  |
| **Hemoglobin** | ≥100 g/L | 170 | 1,7 | 1,2-2,5 | <0,01 | 94 | 1,8 | 1,0-3,4 | 0,055 | 84 | 1,2 | 0,6-2,3 | 0,551 |
|  | <100 g/L | 212 |  |  |  | 108 |  |  |  | 90 |  |  |  |
| **Neutrophils** | ≥1,8×10^9^/L | 345 | 1,8 | 1,1-2,9 | 0,013 | 172 | 2,0 | 1,0-4,0 | 0,056 | 147 | 1,9 | 0,9-4,0 | 0,082 |
|  | <1,8×10^9^/L | 48 |  |  |  | 30 |  |  |  | 27 |  |  |  |
| **Platelets** | ≥100×10^9^/L | 179 | 1,9 | 1,3-2,7 | <0,01 | 88 | 1,3 | 0,7-2,3 | 0,456 | 81 | 1,1 | 0,6-1,9 | 0,864 |
|  | <100×10^9^/L | 204 |  |  |  | 114 |  |  |  | 98 |  |  |  |
| **Bone**  **marrow**  **blasts** | <5% | 227 | RG |  | <0,01 | 110 | RG |  | <0,01 | 106 | RG |  | <0,01 |
|  | 5-10% | 71 | 2,7 | 1,7-4,2 | <0,01 | 38 | 2,6 | 1,2-5,5 | 0,015 | 36 | 3,3 | 1,6-7,1 | <0,01 |
|  | 11-20% | 51 | 2,4 | 1,4-4,1 | <0,01 | 29 | 2,6 | 1,2-5,7 | 0,016 | 27 | 6,3 | 3,0-13,4 | <0,01 |
|  | 21-30% | 16 | 7,5 | 3,5-16,2 | <0,01 | 8 | 5,7 | 1,5-21,5 | <0,01 | 5 | 4,8 | 1,0-23,4 | 0,050 |
|  | >30% | 42 | 5,7 | 3,4-9,7 | <0,01 | 17 | 4,0 | 1,6-9,9 | <0,01 | 0 | n.a. |  |  |
| **Aberrations**  **per case** | 0 | 260 | RG |  | <0,01 | 99 | RG |  | <0,01 | 89 | RG |  | 0,662 |
|  | 1 | 126 | 1,5 | 0,9-2,1 | 0,061 | 60 | 2,8 | 1,3-5,7 | <0,01 | 52 | 1,4 | 0,6-3,1 | 0,386 |
|  | 2 | 31 | 1,7 | 0,9-3,2 | 0,125 | 15 | 0,8 | 0,2-3,1 | 0,763 | 12 | 0,9 | 0,3-3,5 | 0,933 |
|  | 3 | 14 | 6,7 | 3,1-14,2 | <0,01 | 7 | 5,7 | 1,5-21,2 | <0,01 | 6 | 1,3 | 0,3-6,4 | 0,721 |
|  | >3 | 62 | 9,4 | 6,0-14,5 | <0,01 | 21 | 8,3 | 2,5-28,2 | <0,01 | 15 | 2,2 | 0,5-9,3 | 0,270 |
| **IPSS**  **Cytog. score** | favorable | 304 | RG |  | <0,01 |  |  |  |  |  |  |  |  |
|  | intermediate | 84 | 1,8 | 1,1-2,7 | <0,01 |  |  |  |  |  |  |  |  |
|  | unfavorable | 105 | 5,3 | 3,7-7,6 | <0,01 |  |  |  |  |  |  |  |  |
| **IPSS-R**  **Cytog. score** | very good | 11 | RG |  | <0,01 |  |  |  |  |  |  |  |  |
|  | good | 306 | 0,9 | 0,3-2,5 | 0,859 |  |  |  |  |  |  |  |  |
|  | intermediate | 74 | 1,4 | 0,5-4,0 | 0,540 |  |  |  |  |  |  |  |  |
|  | unfavorable | 40 | 3,5 | 1,2-10,2 | 0,024 |  |  |  |  |  |  |  |  |
|  | very unfavorable | 61 | 8,4 | 2,9-23,9 | <0,01 |  |  |  |  |  |  |  |  |
| **CE** | no | 421 | 2,7 | 1,9-4,0 | <0,01 | 153 | 3,6 | 1,3-9,8 | 0,013 | 135 | 4,6 | 1,6-13,1 | <0,01 |
|  | yes | 72 |  |  |  | 47 |  |  |  | 39 |  |  |  |
| **CE**  **patterns** | A | 73 | RG |  | <0,01 | 59 | RG |  | 0,055 | 52 | RG |  | 0,415 |
|  | B | 126 | 1,1 | 0,6-1,8 | 0,831 | 104 | 2,0 | 0,9-4,2 | 0,081 | 87 | 1,0 | 0,5-2,1 | 0,941 |
|  | C | 18 | 0,9 | 0,3-2,3 | 0,801 | 15 | 0,4 | 0,1-1,4 | 0,143 | 15 | 1,0 | 0,3-3,5 | 0,939 |
|  | D | 33 | 11,3 | 5,7-22,2 | <0,01 | 17 | 1,9 | 0,6-6,5 | 0,295 | 14 | 0,2 | 0,0-1,0 | 0,057 |
|  | E | 8 | 0,3 | 0,0-2,6 | 0,305 | 7 | 0,6 | 0,1-5,3 | 0,665 | 6 | 1,3 | 0,3-6,4 | 0,757 |

**Legend:**

CE: clonal evolution; OS: overall survival; LFS: leukemia-free survival; CI: confidence interval; HR: hazard ratio; Cytog. Score: Cytogenetic score; RG: reference group; n: number; p: P-value; OS: Overall survival; LFS: Leukemia-free survival
