# Supplementary figures and images for: Detailed analysis of clonal evolution and cytogenetic evolution patterns in patients with myelodysplastic syndromes (MDS) and related myeloid disorders
Source: Blood Cancer J. 2018 Mar 7;8(3):28. doi: 10.1038/s41408-018-0061-z (PMC5841340; doi:10.1038/s41408-018-0061-z)

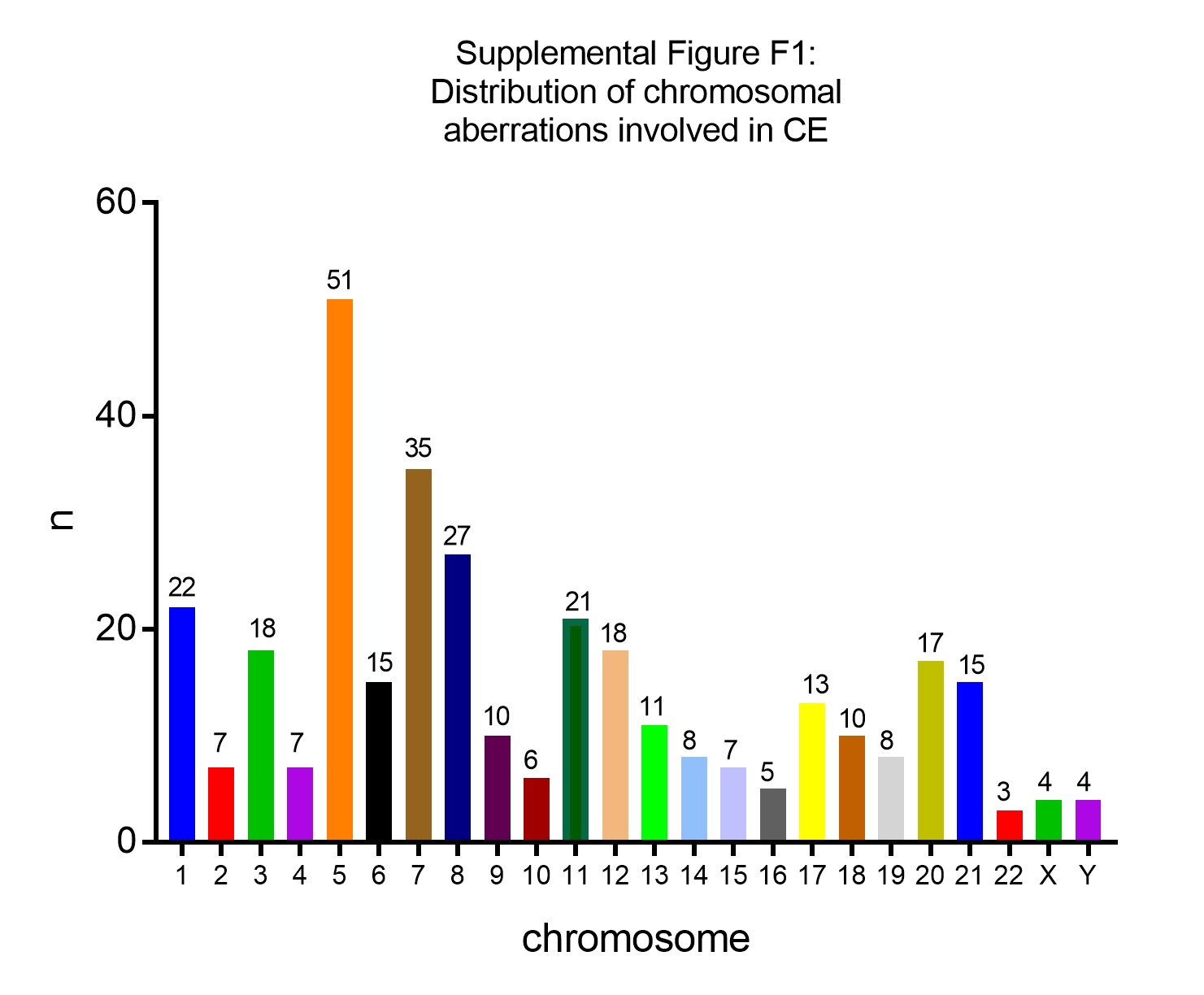

Supplement: Supplementary file 4 — Supplementary Figure 1 [file 41408_2018_61_MOESM4_ESM.tif]

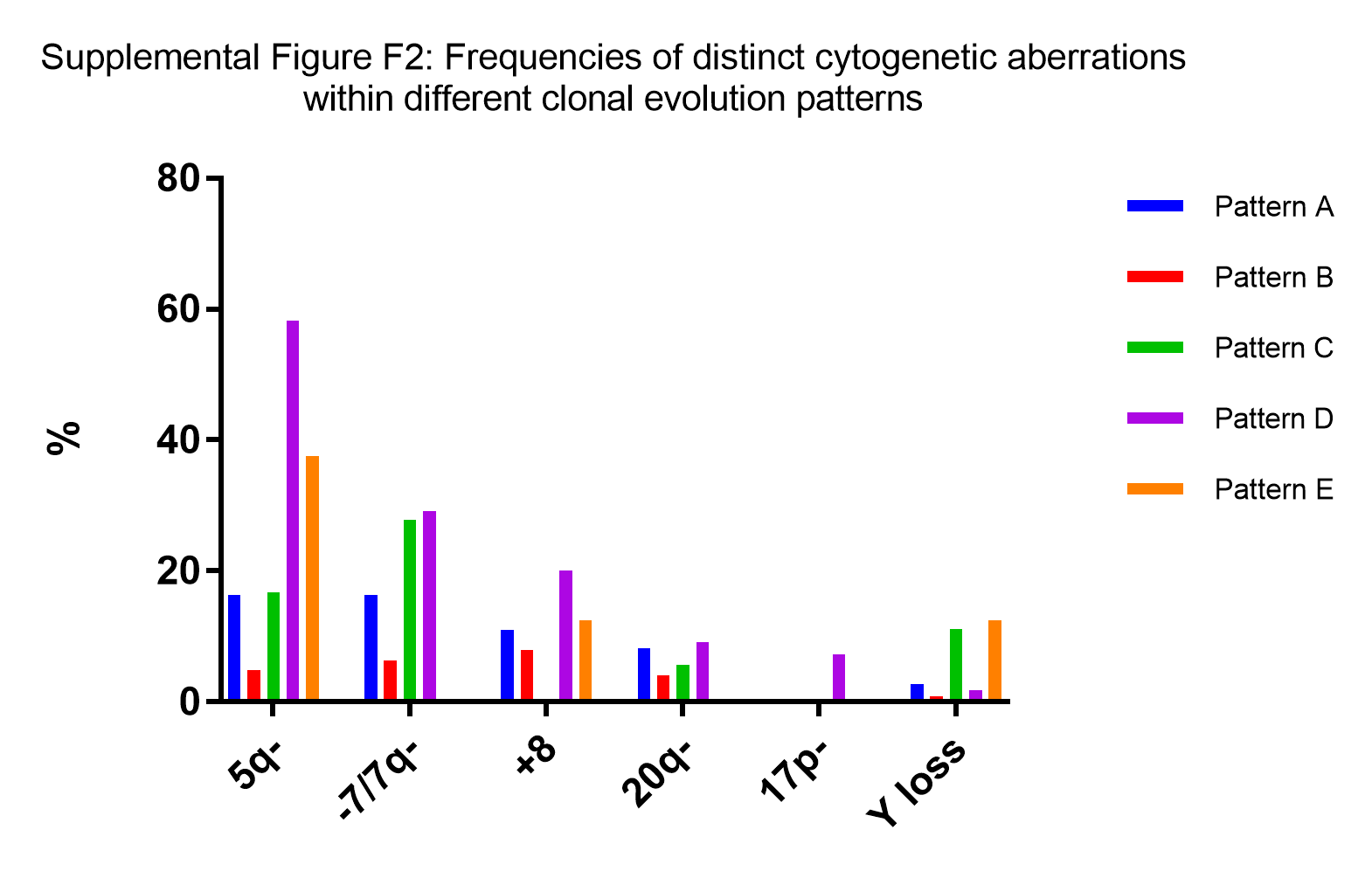

Supplement: Supplementary file 5 — Supplementary Figure 2 [file 41408_2018_61_MOESM5_ESM.tif]
